# Spirulina (*Arthrospira platensis*) Improved Nonalcoholic Fatty Liver Disease Characteristics and Microbiota and Did Not Affect Organ Fibrosis Induced by a Fructose-Enriched Diet in Wistar Male Rats

**DOI:** 10.3390/nu16111701

**Published:** 2024-05-30

**Authors:** Nicole Fakhoury-Sayegh, Aya Hamdan, Sarah Lebbos, Tarek Itani, Viviane Trak-Smayra, Aline Khazzaka, Carole Dagher-Hamalian, Lea Nicole Sayegh, May Mallah, Omar Obeid, Raymond Sayegh

**Affiliations:** 1 Department of Nutrition, Faculty of Pharmacy, Saint Joseph University, Damascus Street, Beirut P.O. Box 11-5076, Lebanon; sarahlebbos@gmail.com; 2 Department of Human Nutrition, College of Health Sciences, QU-Health, Qatar University, Doha 2713, Qatar; ayahamdan@qu.edu.qa; 3 Laboratory of Enteric Virus Infections, Federal Budgetary Institution of Science Federal Scientific Research Institute of Viral Infections «Virome», Federal Service for Surveillance on Consumer Rights Protection and Human Wellbeing, 620030 Yekaterinburg, Russia; itani_tm@niivirom.ru; 4 Department of Pathology, Faculty of Medicine, Saint Joseph University, Damascus Street, Beirut P.O. Box 11-5076, Lebanon; viviane.traksmayra@usj.edu.lb; 5 Department of Surgical Research, Faculty of Medicine, Saint Joseph University, Damascus Street, Beirut P.O. Box 11-5076, Lebanon; aline.khazzaka@usj.edu.lb; 6 Department of Pathology, Gilbert and Rose-Marie Chagoury School of Medicine, Lebanese American University, Byblos P.O. Box 36, Lebanon; carole.hamalian@lau.edu.lb; 7 Department of Gastroenterology and Hepatology, Mayo Clinic, Rochester, MN 55902, USA; sayegh.lea@mayo.edu; 8 Department of Microbiology Research, Faculty of Pharmacy, Saint Joseph University, Damascus Street, Beirut P.O. Box 11-5076, Lebanon; may.mallahhamdan@usj.edu.lb; 9 Department of Nutrition and Food Sciences, Faculty of Agricultural and Food Sciences, American University of Beirut, Beirut P.O. Box 11-0236, Lebanon; omar.obeid@aub.edu.lb; 10 Department of Gastroenterology, Faculty of Medicine, Saint Joseph University, Damascus Street, Beirut P.O. Box 11-5076, Lebanon; raymond.sayegh@usj.edu.lb

**Keywords:** spirulina, fructose, triglycerides, intestinal microbiota, NAFLD

## Abstract

Spirulina (*Arthrospira platensis*) is reported to play a role in improving nonalcoholic fatty liver disease (NAFLD) and intestinal microbiota (IM). To study spirulina’s effects in the improvement of NAFLD characteristics, IM, and pancreatic–renal lesions induced by a fructose-enriched diet, 40 Wistar healthy male rats, weighing 200–250 g, were randomly divided into four groups of 10, and each rat per group was assigned a diet of equal quantities (20 g/day) for 18 weeks. The first control group (CT) was fed a standardized diet, the second group received a 40% fructose-enriched diet (HFr), and the third (HFr-S5) and fourth groups (HFr-S10) were assigned the same diet composition as the second group but enriched with 5% and 10% spirulina, respectively. At week 18, the HFr-S10 group maintained its level of serum triglycerides and had the lowest liver fat between the groups. At the phylae and family level, and for the same period, the HFr-S10 group had the lowest increase in the *Firmicutes/Bacteroidetes* ratio and the *Ruminococcaceae* and the highest fecal alpha diversity compared to all other groups (*p* < 0.05). These findings suggest that at a 10% concentration, spirulina could be used in nutritional intervention to improve IM, fatty liver, metabolic, and inflammatory parameters associated with NAFLD.

## 1. Introduction

Nonalcoholic fatty liver disease (NAFLD) is the world’s most common chronic liver disease [1]. This disease may induce a broad spectrum of liver damage, whereby fatty liver progresses to nonalcoholic steatohepatitis (NASH), cirrhosis, and, ultimately, hepatocellular carcinoma (HCC) [2]. Its diagnosis is based on several parameters such as biological markers, evidence of hepatic steatosis by imaging or histology, and the exclusion of secondary causes of hepatic steatosis such as alcohol consumption and other etiological factors [3,4]. NAFLD is not only a hepatic pathology but a multisystem disease with extrahepatic manifestations [5]. It is linked to other morbidities, including obesity, type 2 diabetes mellitus (T2DM), kidney and cardiovascular disease, and malignant tumors, presumably leading to higher mortality rates [6]. In 2022, a group of panelists decided that metabolic dysfunction–associated steatotic liver disease (MASLD) would replace NAFLD as the preferred term to describe patients with hepatic steatosis and metabolic risk factors, but allowing a moderate amount of alcohol (≤50 g/day for women and ≤60 g/day for men) [7]. Metabolic dysfunction–associated steatohepatitis (MASH) would replace NASH to describe patients with MASLD and active necroinflammation characterized by the presence of lobular inflammation and hepatocyte ballooning [7]. A new entity is metabolic dysfunction and alcohol-associated liver disease, abbreviated as MetALD, which includes patients with MASLD together with moderate alcohol consumption (30 g per day in men, 20–50 g per day in women) [7]. Patients diagnosed with NAFLD or MASLD are twice as likely to develop chronic kidney disease [8]. They also have a high pancreatic fat content, a condition called “fatty nonalcoholic pancreas disease” [9].

Intestinal microbiota (IM) is a metabolic organ composed of 10^14^ microorganisms living mainly in the colon [10]. Recent studies have shown that in addition to genetic predisposition and diet, the gut microbiota affects hepatic carbohydrate and lipid metabolism as well as influences the balance between proinflammatory and anti-inflammatory effectors in the liver, thereby impacting NAFLD and its progression to NASH [11]. IM dysbiosis contributes to the alteration of intestinal permeability, the metabolism of biliary acids, and the production of ethanol [10].

Diet composition is an environmental factor that might influence NAFLD severity. A healthy diet such as a reduction in caloric intake and high-glycemic index (GI) foods and an increased consumption of monounsaturated fatty acids, omega-3 fatty acids, fiber, and specific protein sources such as fish and poultry are suggested to have beneficial effects on fatty liver [12]. On the contrary, a high-sugar diet, mainly a high-fructose diet (30–40% of fructose of total energy intake (TEI)), has been found to play an important role in the development of this disease [13], as well as in the modification of the IM, knowing that the latter is the interface between diet and the liver [14].

Spirulina (*Arthrospira platensis*) is a spiral-shaped, filamentous, photosynthetic cyanobacterium/alga that blooms well in alkaline waters [15,16,17]. This blue-green alga has recently received a lot of attention for its role in the treatment of NAFLD. Spirulina supplementation has been shown to correlate with its improvement and to prevent fructose-induced fatty liver [15,18,19,20]. Several studies have shown the inverse effect of a diet supplemented with 5% and 10% spirulina on serum aspartate aminotransferase (AST), alanine aminotransferase (ALT) levels, as well as triacylglycerols and fatty liver grades [20,21].

However, research on the protective effects of spirulina in improving kidney and pancreas function and IM induced by a 40% fructose-enriched diet is lacking. Some studies showed promise in improving metabolic disorders like lipid and carbohydrates metabolism, particularly in high-sucrose or high-fat diet scenarios [22,23]. However, more studies, with different diet compositions, are needed to provide deeper insights into spirulina’s potential benefits for kidney and pancreas health and microbiota.

Thus, the objective of the present study was to investigate the effects of 5% and 10% spirulina added to a 40% fructose-enriched diet on the improvement of NAFLD characteristics, on pancreatic–renal lesions in Wistar male rats, microbiota composition, and its diversity. The hypothesis was that 5% and 10% spirulina added to a fructose-enriched diet would protect these organs from lesions and damage, maintain gut microbiota integrity, and enhance NAFLD characteristics.

## 2. Methods and Materials

### 2.1. Experimental Design and Animals

Forty pathogen-free Wistar rats (males, 6 months old), weighing between 200–250 g, were housed individually in chip-bedded plastic cages (50 × 50 cm) in a controlled temperature (23 ± 2 °C) and humidity (60 ± 10%) under a 12:12-h diffuse light/dark cycle in the Surgical Research Laboratory of Saint Joseph University for 18 weeks. All rats were from pure Wistar crosses with the absence of ob/ob gene mutations [13]. Rat cages conformed to the Animal Research Review Panel (ARRP) guidelines [24]. All rats received human care and had visual, auditory, and olfactory contact with each other.

### 2.2. Diets and Blood Collection

At week 1, all rats received a chow diet (16% proteins, 3% fat, and 60% carbohydrates) ad libitum. At the end of week 1, the rats were starved for 16 h before the collection of 2 mL of blood from the jugular vein, under general anesthesia (0.2 mL per rat of a mixture comprising 10 mL of 50 mg/mL ketamine and 2 mL of 25 mg/mL xylazine, injected intramuscularly) [13].

At week 2, the 40 rats were divided randomly into four groups (10 rats per group). The first group received 20 g of a standardized diet (CT) (15.61 kJ/g; 17% of fats, 20% of proteins, and 62% of carbohydrates). The second group received a 40% fructose-enriched diet (HFr) (15.9 kJ/g; 20% fats, 20% proteins, 60% carbohydrates). The third and fourth groups received the same diet composition as the second group with 5% and 10% spirulina of total weight, respectively (HFr-S5 and HFr-S10) (Table 1). The fat used in the last three diets consisted of butter (51% saturated fatty acid, 21% monounsaturated fatty acid, and 3% polyunsaturated fatty acid) and soya bean oil (16% saturated fatty acid, 23% monounsaturated fatty acid, and 58% polyunsaturated fatty acid) [13]. Twenty grams of food per rat per day was considered the standard amount of food used by rats per day [25]. All rats had free access to drinking water in a feeding bottle.

The rat diet was prepared each month (at Saint Joseph University) by following the American Institute of Nutrition-93G diet [26] and stored at a temperature of 3–4 °C. The rat’s weight and the food intake per rat per day were measured on a daily and weekly basis. The absolute amount of food consumed per rat per day was changed to energy intake (kJ per rat per week) and was calculated by subtracting the 20 g of food given per rat per day from the amount left or spilled in the cage [13].

At week 18, all rats were starved for 16 h before excising and weighing their livers under general anesthesia (0.2 mL per rat of a mixture comprising 10 mL of 120 mg/kg ketamine and 2 mL of 15 mg/kg xylazine, injected intramuscularly). Two milliliters of blood sample per rat were simultaneously obtained from the inferior vena cava. The kidneys, epididymal white fat, and pancreas were also removed and weighed [13].

### 2.3. Preparation of Spirulina (A. platensis)

Spirulina *(A. platensis)* used in the experimental diet was purchased from General Nutrition Care (GNC), Beirut, Lebanon, in the form of blue-green capsules. In the process of the preparation, the capsules were unsealed and the powder was blended with the diet until a fully homogeneous mixture.

### 2.4. Sample Preparation, Histological Examination of the Liver, Kidney, and Pancreas; and Liver and Kidney Lipid Determination

Plasma was separated by centrifugation (1000× *g*; 5 min) immediately after collection and stored at −80 °C until analysis. Fragments of the rat’s liver, kidney, and pancreas were selected randomly, removed, and sent to the pathology laboratory for histological examination. The liver fragments were fixed in 10% formalin, routinely processed, and embedded in paraffin. Sections of 3 μm were cut from the paraffin block and stained with hematoxylin and eosin (H&E). Picrosirius red staining was performed to evaluate the degree of portal and peri-sinusoidal fibrosis in the tissue. Frozen liver sections were used to evaluate steatosis by staining with oil-red O [27].

An optic microscopy (Axioskop, Zeiss, Oberkochen, Germany) was used to examine the slides. The percentage of steatosis was determined by evaluating the number of fat-enriched hepatocytes, multiplied by 100, over the total number of cells in 10 randomly chosen different medium-power fields (200×). The pattern of lipid accumulation in hepatocytes was also studied. Microvesicular and macrovacuolar steatosis patterns, necroinflammation, portal fibrosis, and perisinusoidal fibrosis were scored as absent (0), mild (1), or moderate (2), depending on the percentage of fatty hepatocytes, the number of inflammatory infiltrates, and the extent of extracellular matrix deposition. Results for macrovacuolar and microvesicular steatosis were given as the number of rats presenting as mild (+; <33% of hepatocytes) or moderate (++; 33–66% of hepatocytes) [28]. Mild necroinflammation refers to a few lobular aggregates of inflammatory cells with or without apoptotic bodies. Necroinflammation was considered moderate when at least one lobular area contained two or more such aggregates [13].

For the scoring, the kidney and pancreas samples were fixed in 10% formalin, routinely processed, and stained with H&E and Masson’s trichrome stains. Inflammation in the kidney and pancreas, interstitial renal fibrosis, glomerulosclerosis, and pancreatic tissue fibrosis were scored as absent (0), mild (1), or moderate (2). Pancreatic islets of Langerhans were studied to evaluate any abnormalities such as hyperplasia (increased islet size by multiplication of cells) or hypertrophy (increased size related to the increased volume). The scoring of various organ groups was performed in a blinded manner to ensure a high level of rigor [13].

### 2.5. Liver and Kidney Lipid Extraction

Forty frozen fragments of liver and kidney tissue from the 40 rats were also randomly sectioned to determine the total lipid content per rat. The liver and kidney samples were weighed before and after freeze drying (2.5 Liter Bench Top Freeze-Dry System, LABCONCO, Kingston, NY, USA). After freeze drying for 24 h, the sections were crushed and the samples were placed in moisture-free sealable filter bags and weighed before and after lipid extraction. The lipids in the samples were extracted for 40 min per run using petroleum ether solvent (BP 400–600 °C in an Ankom XT10 extractor, (Ankom Technology, Macedon, NY, USA). The lipid weight was determined by subtracting the weight difference of the samples before and after lipid extraction [13,29,30].

### 2.6. Serum Chemistry

A commercially available kit (Trinder method, Biolabo SA, Maizy, France) and a spectrophotometer were used to measure the serum levels of fasting glucose and triglycerides (TG) [13]. Serum creatinine and urea were also measured with commercially available kits (Auto-Creatinine liquicolor, urea kit, Human, Munich, Germany) and a spectrophotometer. ALT and AST were analyzed using ELISA kits (Rat Alanine Aminotransferase, and Rat Aspartate Aminotransferase (Antibodies Online, Aachen, Germany) Serum tumor necrosis factor (TNF-α), interleukine-6 (IL-6), and adiponectin levels were analyzed using ELISA kits (Rat High Sensitive, R&D Systems, Minneapolis, MN, USA). Serum insulin concentrations were also analyzed using ELISA kits (rat insulin) (Antibodies Online, Aachen, Germany) [13].

### 2.7. Fecal Microbiome Analyses

Fecal samples were collected from Wistar rats at the beginning of this study (day 1) and at the end of week 18. They were stored at −80 °C until their microbial analyses. Fecal total microbial DNA was extracted from the fecal samples using a DNA isolation kit according to the manufacturer ‘s protocols (QIA amp DNA Stool Mini Kit, Qiagen, Hilden, Germany). The isolated DNA served as template for PCR originating from DNA barcoded universal 16SrRNA gene primers that amplified the V3 and V4 variable regions of the 16S rRNA gene [31].

The microbial community was assessed by high-throughput sequencing of the bacterial 16S rRNA gene through the GeT-PlaGe platform in INRAE, Toulouse, France using Illumina MiSeq technology. The V3V4 region was amplified from purified DNA with the primers F343 (CTTTCCCTACACGACGCTCTTCCGATCTTACGGRAGGCAGCAG) and R784 (GGAGTTCAGACGTGTGCTCTTCCGATCTTACCAGGGTATCTAATCCT) using 30 amplification cycles with an annealing temperature of 65 degrees (an amplicon of 510 bp). Single multiplexing was performed using a homemade 6 bp index, which was added to R784 during a second PCR with 12 cycles using forward primer (AATGATACGGCGACCACCGAGATCTACACTCTTTCCCTACACGAC) and reverse primer (CAAGCAGAAGACGGCATACGAGAT-index-GTGACTGGAGTTCAGACGTGT). The resulting PCR products were purified and loaded onto Illumina MiSeq cartridges (San Diego, CA, USA) and sequenced on an Illumina MiSeq instrument with 2×300 paired-end read sequencing according to the manufacturer’s instructions. The quality of the run was checked internally using PhiX, and then each pair-end sequence was assigned to its samples with the help of the previously integrated index. Each pair-end sequence was assembled using Flash software, version 1.2.11 (Magoc 2011) using at least a 10 bp overlap between the forward and reverse sequences, allowing a 10% mismatch. The lack of contamination was checked with a negative control during the PCR using water as a template. The quality of the stitching procedure was controlled using four bacterial samples that were run routinely in the sequencing facility in parallel to the current samples.

Sequences were analyzed and normalized with the pipeline FROGS (Find Rapidly Operational Taxonomic Units (OTUs) with Galaxy Solution) [32]. PCR primers were removed, and sequences with sequencing errors in the primers were excluded. Reads were clustered into OTUs using the swarm clustering method. Chimeras were removed, and 1038 OTUs were assigned at different taxonomic levels (from phylum to species) using the RDP classifier and NCBI Blast+ on the Silva_123_16S database.

The sequences were aligned using Clustal Omega 1.1.0 with the profile alignment option in Sea View 4.5 [33]. Neighbor joining trees as well as maximum-likelihood trees using PhyML 3.1 were built to assess identifications [34].

The microbiota of all rat groups was analyzed using high-throughput sequencing. Microbial diversity analyses were performed by clustering sequence tags into groups of defined sequence variation. α-diversity measurements (observed OTUs, Chao 1, Shannon diversity index or SDI and inverted Simpson index) and β-diversity measurements (Jaccard, Bray-Curtis, UniFrac and weighted UniFrac) were analyzed using a blocked analysis of variance. The relative abundance of bacteria was compared with a MULTINOVA using the Jaccard and unweighted UniFrac similarity measures to construct distance metrics. All analyses were conducted using the R programming language in FROGS.

### 2.8. Ethical Considerations

All experiments took place at the Surgical Research Laboratory of Saint Joseph University Medical School (Beirut, Lebanon) in accordance with the “Guide for care and use of laboratory animals” (Department of Health and Human Services. Public Health Service, National Institutes of Health. NIH Publication No. 86-23, Revised 1985). The protocol was accepted by the ethical committee of the Medical School of Saint Joseph University, Beirut, Lebanon (USJ-341).

### 2.9. Statistical Analyses

The sample size of 40 rats corresponds to the minimal size recommended for experimental animal studies to detect significant differences among the four groups with a 95% confidence interval and a power of 80% [25,35]. Continuous variables were expressed as the means ± standard deviation (SD). Geometric means (Log 10 of continuous variables) were used in the case of nonnormality of distribution. Statistical analyses were performed using Student’s paired *t*-test. One-way between-groups analysis of variance (ANOVA) over week 1 and week 18 for normally distributed data was also performed, followed by the Bonferroni multiple comparisons test. The significance level was set at *p* < 0.05. Statistical analysis was performed using SPSS 20 for Windows release (IBM Corp. Released 2011. IBM SPSS Statistics for Windows, Version 20.0. Armonk, NY, USA).

## 3. Results

### 3.1. Body Weight (g), Energy Intake (kJ/Week), Amount of Food Consumed (g/Rat/Week), and Organ Weights (g)

At the end of week 18, there were no significant differences among the four groups in terms of the rat’s body weight (g). Within the same group, the HFr group showed a significant increase in energy intake (kJ/week) and the amount consumed (g/week) between week 2 and week 18 (*p* < 0.05).

At week 18, the liver weight (g) was significantly higher in the HFr group compared to the CT group (*p* < 0.05) (Table 2). No significant differences were observed among the four groups for the kidney, pancreas, and epididymal fat weight (Table 2). Liver lipid weight (mg) was significantly higher in the HFr group compared to the HFr-S10 group (*p* < 0.05). No significant differences were observed among the four groups for the renal lipid weight (mg) (Table 2).

### 3.2. Liver Histopathology

**Figure 1:**
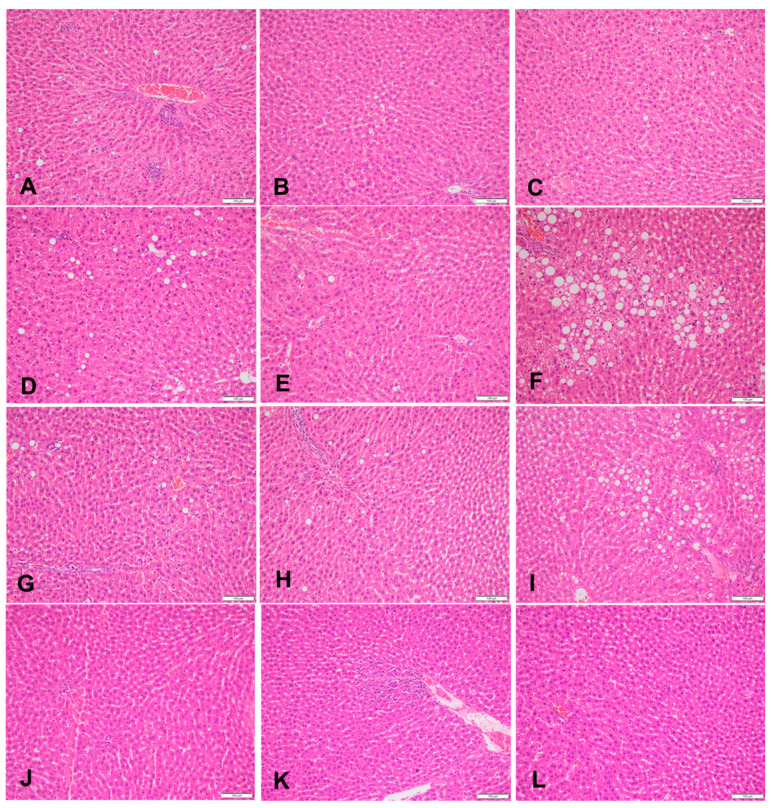
Sections of liver in (**A**–**C**) a rat fed the control diet (H&E, 200×), (**D**–**F**) area of microvesicular steatosis in a rat fed a high-fructose diet (H&E, 200×), (**G**–**I**) sections of liver of a rat fed a high-fructose diet and 5% spirulina (H&E, 200×), and (**J**–**L**) sections of liver of a rat fed a high-fructose diet and 10% spirulina (H&E, 200×).

At week 18, the HFr group showed the highest percentage of steatosis (10.67% ± 0.24%) whereas the HFr-S10 group had the lowest percentage of steatosis (1.33% ± 0.13%). In the HFr group, 30% of the rats had mild microvesicular steatosis and 40% had moderate microvesicular steatosis (Table 3, Figure 1D–F). Regarding macrovacuolar steatosis, 20% of the HFr group had moderate macrovacuolar steatosis versus none in both the control and HFr-S10 groups (Table 3). Mild necroinflammation was observed in 80% of the rats in the HFr group, whereas in the HFr-S5 and HFr-S10 groups, the proportions were 60% and 40%, respectively. However, moderate necroinflammation was observed in 10% of the HFr-S5 and HFr-S10 groups. Mild portal fibrosis was present in a proportion of 10% in the HFr and HFr-S10 groups and in a proportion of 20% in the HFr-S5 group (Table 3). Mild perisinusoidal fibrosis was present in a proportion of 10% in the first three groups and in a proportion of 20% in the HFr-S10 group (Table 3).

### 3.3. Histopathology of the Kidneys and Pancreas

**Figure 2:**
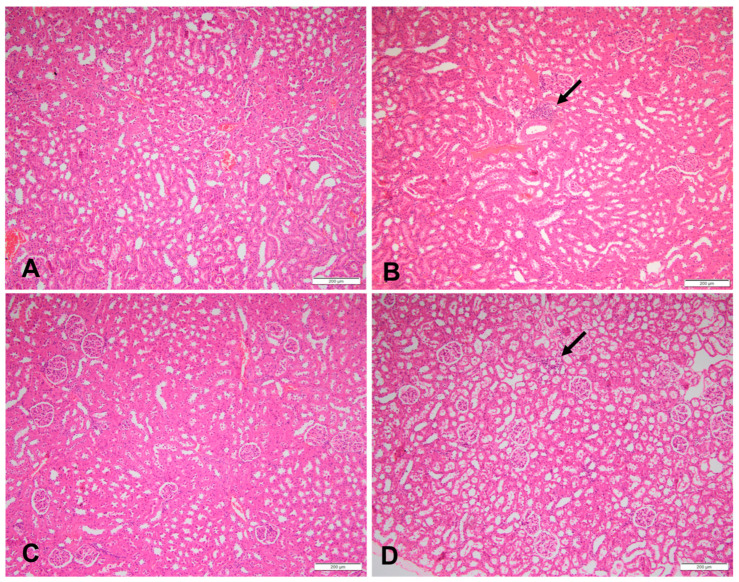
Sections of a kidney in (**A**) a rat fed the control diet (H&E, 100×), (**B**) area of inflammation in a rat fed a high-fructose diet (the arrow) (H&E, 100×), (**C**) sections of a kidney of a rat fed a high-fructose diet and 5% spirulina (H&E, 100×), and (**D**) sections of a kidney with an area of inflammation (the arrow) in a rat fed a high-fructose diet and 10% spirulina (H&E, 100×).

**Figure 3:**
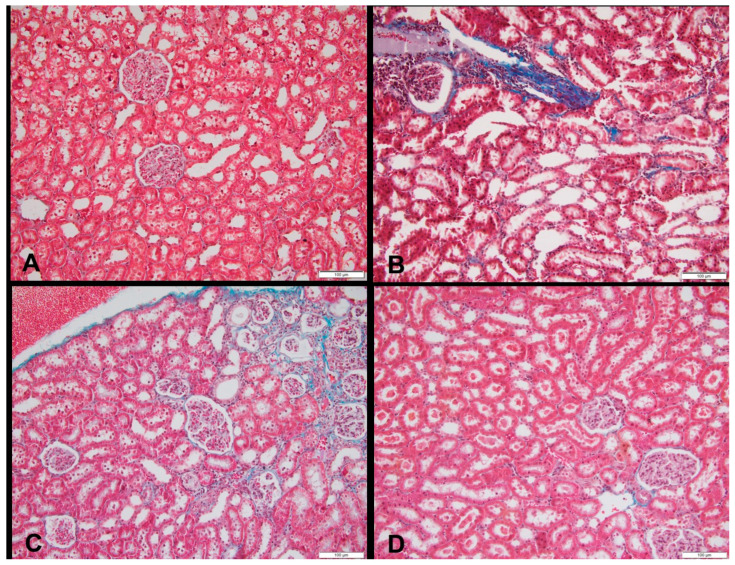
Sections of a kidney in (**A**) a rat fed the control diet (Masson’s trichrome, 200×), (**B**) area of fibrosis in a rat fed a high-fructose diet (blue color), (Masson’s trichrome, 200×), (**C**) area of fibrosis in a rat fed a high-fructose diet and 5% spirulina (blue color) (Masson’s trichrome, 200×), and (**D**) sections of a kidney with an area of fibrosis in a rat fed a high-fructose diet and 10% spirulina (blue color), (H&E, 100×).

**Figure 4:**
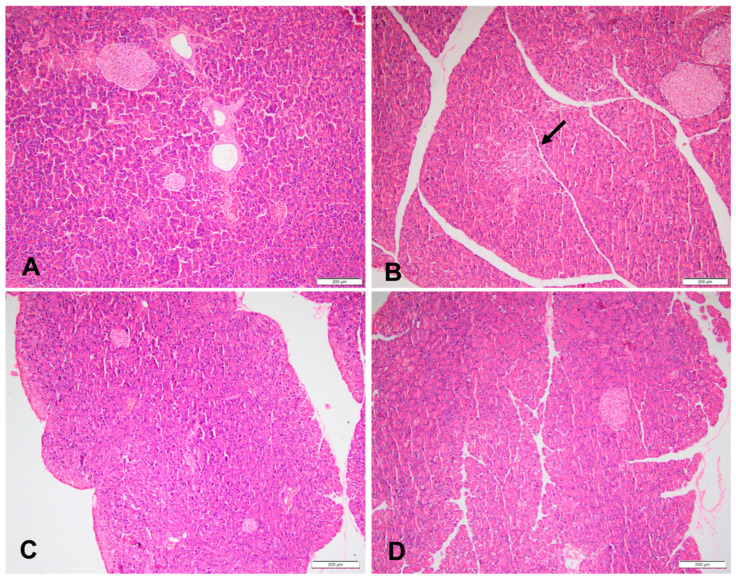
Sections of a pancreas in (**A**) a rat fed the control diet (H&E, 100×), (**B**) area of pancreas inflammation, (the arrow), in a rat fed a high-fructose diet (H&E, 100×), (**C**) sections of a pancreas of a rat fed a high-fructose diet and 5% spirulina (H&E, 100×), and (**D**) sections of a pancreas of a rat fed a high-fructose diet and 10% spirulina (H&E, 100×).

**Figure 5:**
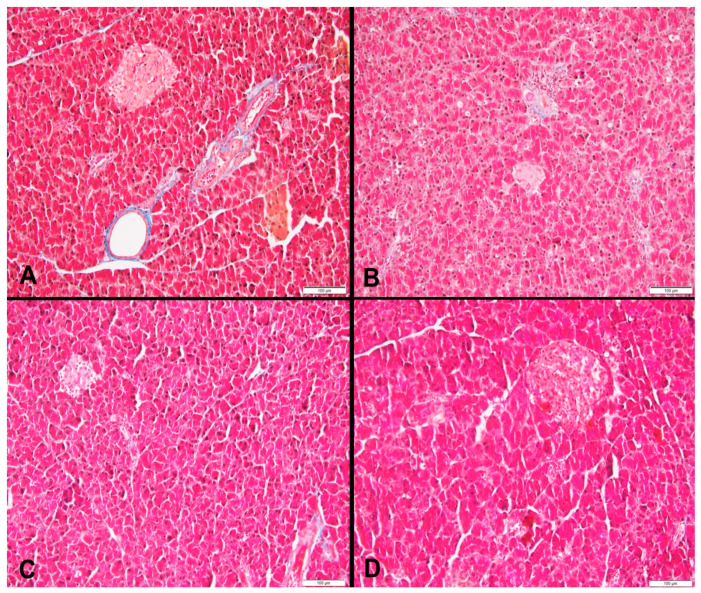
(**A**) Area of pancreas fibrosis in a rat fed the control diet (blue color) (Masson’s trichrome, 200×), (**B**) area of pancreas fibrosis in a rat fed a high-fructose diet (blue color) (Masson’s trichrome, 200×), (**C**) area of pancreas fibrosis in a rat fed a high-fructose diet and 5% spirulina (blue color) (Masson’s trichrome, 200×), and (**D**) area of pancreas fibrosis in a rat fed a high-fructose diet and 10% spirulina (blue color) (Masson’s trichrome, 200×).

At week 18, 60% of the HFr and HFr-S10 rat groups showed mild renal inflammation (60%) compared to the CT and HFr-S5 groups (40% and 10%, respectively), and 10% of the HFr-S10 group showed moderate renal inflammation (Table 4, Figure 2B,D). None of the rat groups showed glomerulosclerosis, and 30% of the CT, HFr, and HFr-S5 groups showed mild interstitial renal fibrosis, compared to 50% in the HFr-S10 group (Table 4, Figure 3B–D). Mild inflammation of the pancreas was observed in 40% of the HFr rat’s group versus 20% in the HFr-S10 group (Table 4, Figure 4B). Moreover, 80% of HFr group showed mild pancreatic tissue fibrosis compared to 50% in the HFr-S10 group, 20% in the HFr-S5 group, and 30% in the control group (Table 4, Figure 5A–D). There was no evidence of prior hyperplasia or hypertrophy in the islets of Langerhans of all groups. The distribution of islets and their size or shape did not change in the studied groups.

### 3.4. Serum Chemistry

At week 18, the fasting serum glucose level was significantly higher in the HFr, HFr-S5, and HFr-S10 groups compared to the CT group, and the mean serum level TG was significantly increased in the HFr group compared to other groups. It also increased significantly between week 1 and week 18. The serum adiponectin level was decreased in the HFr group (week 1–week 18) (Table 5), (*p* < 0.05). Moreover, a significant decrease in the mean of serum TNF-α and in urea level (mmol/L) was observed in the HFr-S10 group (week 1–week 18), (Table 5). No significant differences in the rest of the biological parameters were observed between groups and within groups.

### 3.5. Gut Microbiota Diversity and Composition

**Figure 6:**
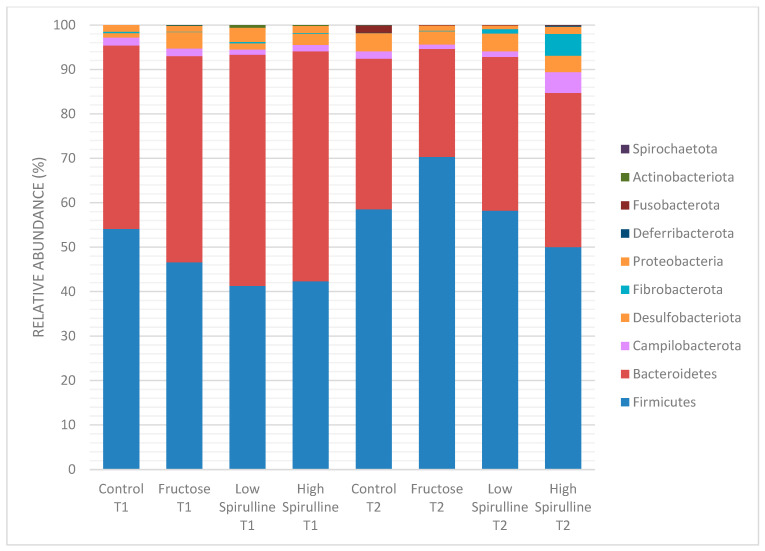
Variation of the different bacterial phyla (%) in each group at time 1 (week 1) and time 2 (week 18). Low spirulina; HFr-S5, High spirulina; HFr-S10.

**Figure 7:**
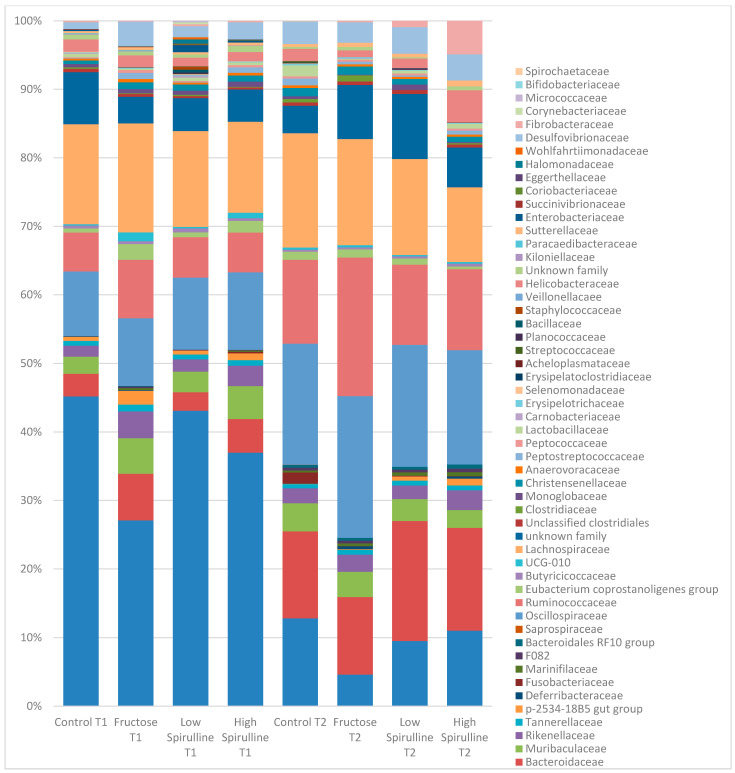
Variation of the different bacterial families (%) in each group at time 1 (week 1) and time 2 (week 18). Low spirulina; HFr-5; High spirulina; HFR-S10.

The taxonomy-based analysis of bacterial families identified the bacterial communities at the two time periods (week 1–week 18). The study results showed the variation of the different bacterial families and phylae between and within groups at the two time periods (Figure 6 and Figure 7).

**Figure 8:**
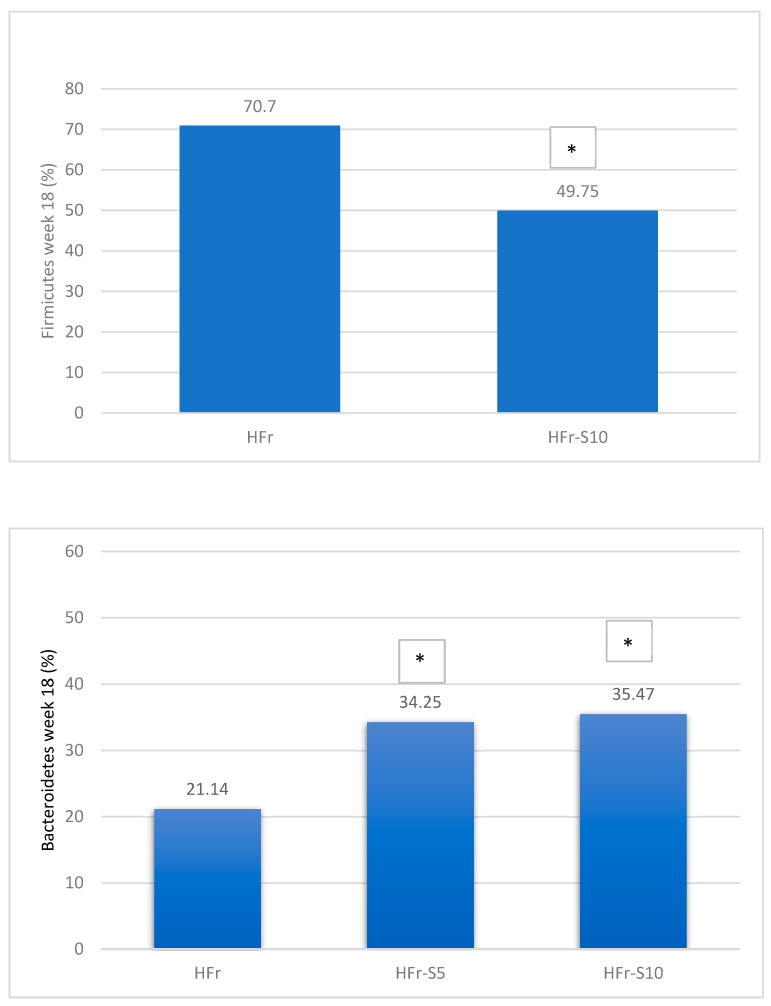

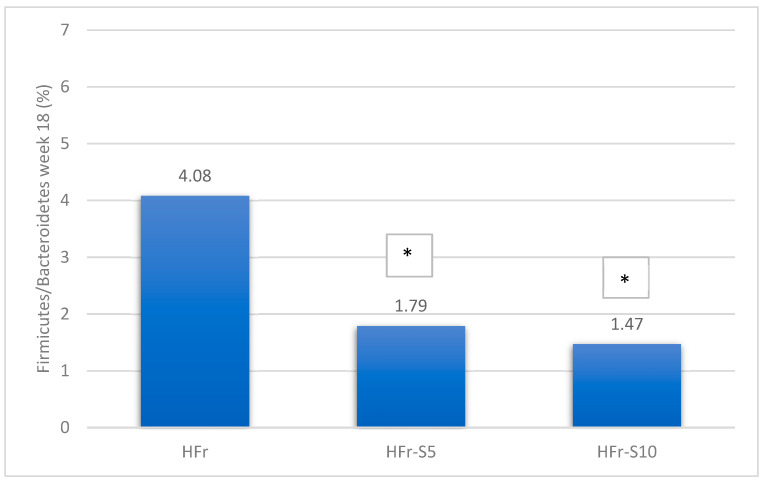
Variation of the mean percentages of the different phylae families (%) between and within groups (week 1–week 18). * *p* < 0.05 when compared with HFr.

A significant decrease in Bacteroidetes phylae was observed in all groups at week 18, mainly in the HFr group (Figure 8). On the other hand, at the same period, the Firmicutes phylae increased significantly in the HFr group compared to other groups (*p* < 0.05) (Figure 8). At week 18, the average ratio of Firmicutes/Bacteroidetes in the HFr group was significantly different from the HFr-S10 groups (Figure 8).

**Figure 9:**
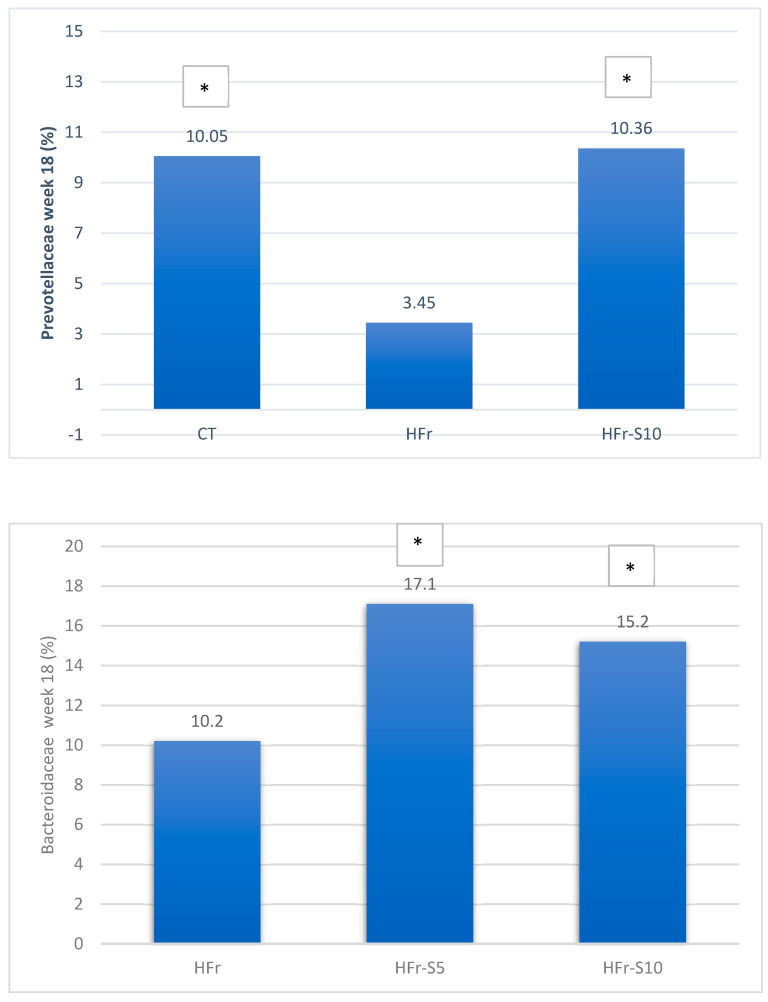

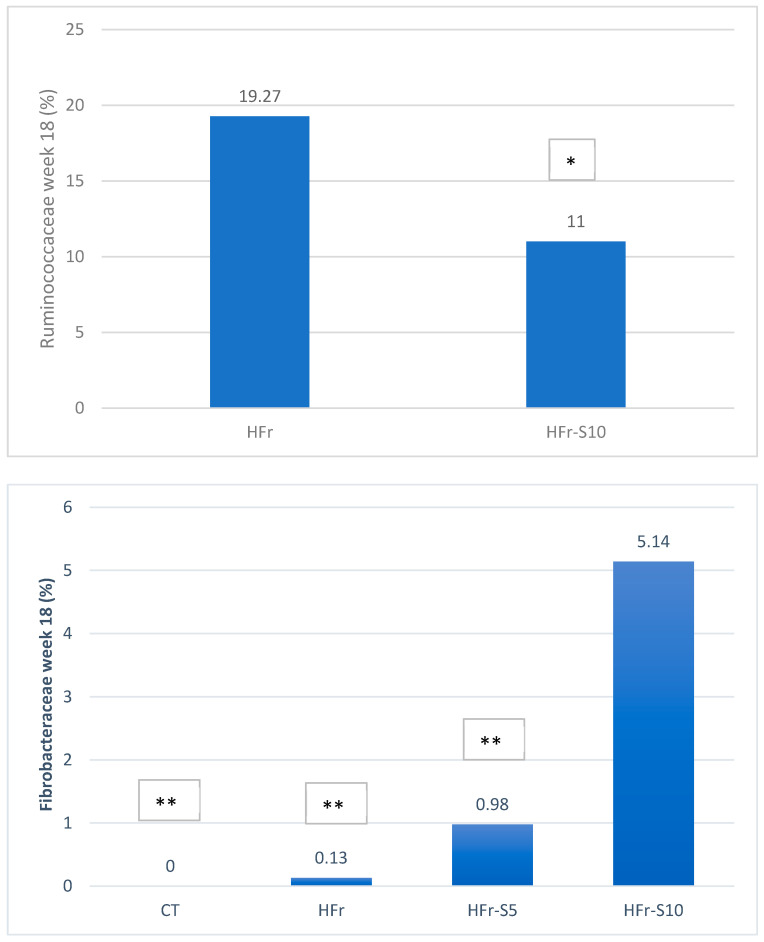
Variation of the mean of the different bacterial families (%) between and within groups (week 1–week 18). * *p* < 0.05 when compared with HFr. ** *p* < 0.05 when compared with HFr-S10.

At week 18, at the family level, the average percentages of *Prevotellaceae* were significantly reduced in the HFr group compared to the HFr-S10 and CT groups (*p* < 0.05), and the family *Bacteroidaceae* showed a significant increase in both groups HFr-S5 and HFr-S10 compared to the HFr group (Figure 9). According to the *Ruminococcoceae* family, the HFr group was significantly higher than the HFr-S10 group at week 18 (Figure 9). Similarly, there was a significant increase in *Fibrobacteraceae* in the HFr-S10 within times and compared to the HFr group at week 18 (Figure 9).

**Figure 10:**
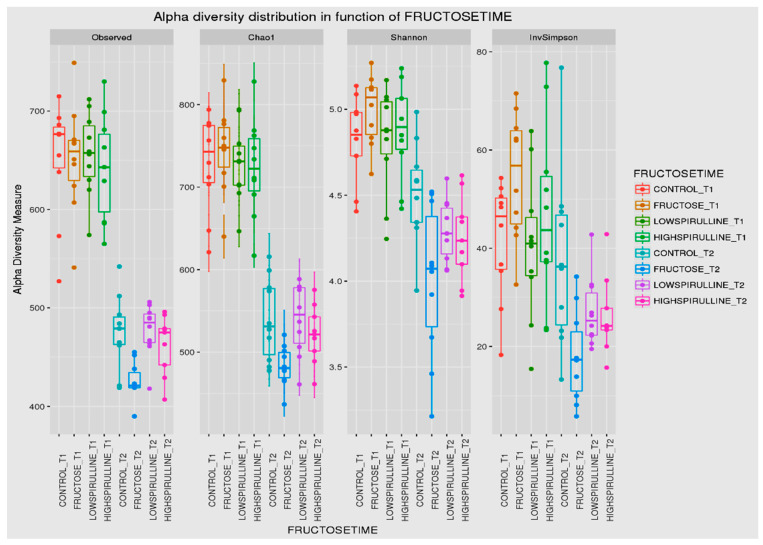
Box plot showing the alpha diversity of the IM within each group between time 1 (week 1) and time 2 (week 18), expressed by the Shannon, Chao 1, and Observed diversity indices.

**Figure 11:**
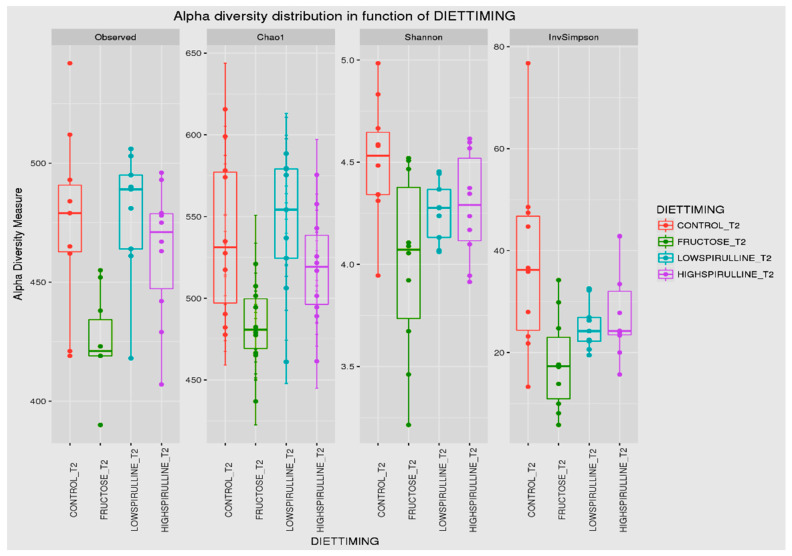
Box plot showing the alpha diversity of the IM at week 18, expressed by the Shannon, Chao 1, and Observed diversity indices.

**Figure 12:**
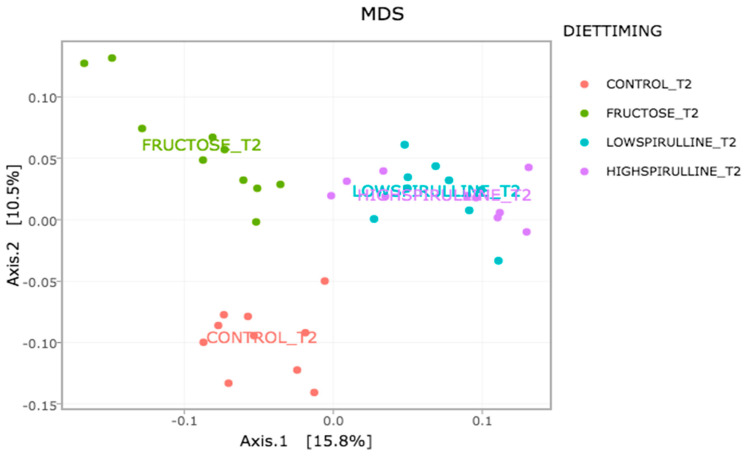
Variation of the composition of the IM between the groups at week 18 according to the Unifrac model.

Major differences in alpha and beta diversity were observed at week 18 between the HFr group and other groups, *p* = 0.0046 (Chao 1) and *p* = 0.0012 (Observed). Statistical analysis indicated that there were significant differences in the alpha diversity between the HFr-S5, HFr-S10, and HFr groups (Figure 10 and Figure 11). The latter showed less diversity than the other groups, *p* < 0.05 (Figure 10 and Figure 11). The HFr-S10 group had even better diversity than the control group (Observed and Chao1) (Figure 10 and Figure 11). The beta diversity showed a greater similarity of the populations of both HFr-S5 and HFr-S10 groups. Both groups had the same bacterial diversity, different from the two other groups (Figure 12).

## 4. Discussion

This study used an animal model consuming a diet enriched in 40% fructose and the same diet but enriched in spirulina at 5% and 10% of its total weight for 18 weeks. Unlike previous studies with ad libitum diets, this study strictly controlled diet amounts to 20 g per rat per day to eliminate calorie excess bias [13,36,37,38].

This study is the first to investigate the effects of spirulina on kidney and pancreas lesions, and on microbiota, caused by a fructose-enriched diet over an extended period.

At week 18, the HFr group exhibited a significant increase in energy intake compared to week 1, likely influenced by fructose’s impact on central appetite regulation. This effect may be attributed to alterations in specific components of the endocannabinoid system, leading to a hunger-like state in the brain [39,40]. Interestingly, our findings on energy intake (kJ) and the amount consumed (g) per week in the HFr-S5 and HFr-S10 groups align with previous studies that have shown that spirulina contains phenylalanine, a potent cholecystokinin stimulant known to suppress appetite by acting on the brain’s appetite center [19,41].

At week 18, the HFr group exhibited the highest liver weight, likely due to the accumulation of lipid vacuole deposits in hepatocytes, resulting in moderate micro- and macrovesicular steatosis. This indicates the accumulation of fatty acids in hepatocytes and their esterification as lipid droplets [25]. Other studies, evaluating the effect of fructose on NAFLD, have shown similar results [25,36,42,43,44]. Fructose acts directly at the hepatic level, promotes lipogenesis, and leads to NAFLD characteristics [45]. In contrast, the other groups presented only mild steatosis except for the HFr-S5 group, which showed 10% moderate micro- and macrovacuolar steatosis (Table 3). In addition, the administration of spirulina at a dose of 10% seemed to attenuate steatosis (1.33%) and the liver fat weight (mg) was significantly lower in the HFr-S10 group compared to the HFr group. Similar results have been found by Pak et al., where the spirulina slowed the development of NASH [46]. Diets enriched with spirulina can improve fatty liver through its antioxidant, lipid-lowering, and anti-inflammatory effects [41,47,48]. The presence of mild necroinflammation in all groups can be attributed to the increased percentage of steatosis as well as the presence of mild micro- and macrovesicular steatosis [25]. In our model, 10–20% of rats, independently of groups, showed mild portal and perisinusoidal fibrosis. This may be due to an excess amount of vitamin A (µg) present in all diets. The concentration of vitamin A (trans-retinyl palmitate) in the AIN-93-VX vitamin mix used was 0.8 g/kg while the nutritional requirement for vitamin A, according to the nutritional requirement of laboratory animals, is 1.3 mg/kg [29]. An increase in hepatic stellate cells, which are vitamin A–storing cells located in Disse’s space around the hepatic sinusoids, can lead to the induction of perisinusoidal fibrosis [13,29].

NAFLD has been linked to extrahepatic morbidity, including pancreatic–renal injury [49]. Data from recent studies in animals and humans suggest that consumption of fructose causes kidney damage and is related to metabolic problems [50].

This study found that 60% of the HFr and HFr-S10 groups showed mild renal inflammation compared to the CT and HFr-S5 groups (Table 4), indicating fructose’s detrimental impact on the kidneys. Fakhoury et al. and De Castro et al. found that rats fed a diet enriched in fructose (60%) exhibited renal damage, increased kidney weight, and inflammation [13,44].

Fructose-induced renal inflammation may occur through various mechanisms, including activation of the polyol pathway, insulin resistance, and stimulation of pro-inflammatory cytokines [13,44,51,52,53]. Although spirulina is recognized for its antioxidant and anti-inflammatory properties, mild renal inflammation persisted in the HFr-S10 group (60% of rats) [54,55,56]. Further investigations, possibly with an extended experimental duration or different rat strains, may be necessary to fully elucidate the effect of a 10% spirulina–enriched diet on renal inflammation.

In our study, we investigated pancreatic injury as an extrahepatic complication of NAFLD, because patients with fatty liver often exhibit increased pancreatic fat content [9]. However, we observed no differences in pancreatic weight among the groups, consistent with findings by De Castro et al. [44]. Targher et al., conversely, have reported a positive association between pancreatic fat accumulation and liver fat content in humans [57].

At week 18, no evidence of hypertrophy and hyperplasia in the islets of Langerhans was found in all rat groups. Mild pancreatic inflammation and tissue fibrosis were observed in all groups but at different rates. The HFr group exhibited the highest percentage of both inflammation and fibrosis compared to other groups (Table 4). Previous research showed that pancreatic inflammation and fibrosis may be related to elevated serum uric acid levels induced by fructose, which stimulates inflammatory mediators and oxidative stress in islet cells [13,57,58,59,60].

Biological parameters such as fasting hypertriglyceridemia, hyperglycemia, and hyperinsulinemia were studied because they represent the main parameters of the metabolic syndrome, with NAFLD considered its hepatic component [61,62]. In the current model, hyperglycemia was observed in all groups at week 1 and week 18, except for the CT group (Table 5). This may be attributable to the anesthetics (ketamine and xylazine) used during the two time periods, especially because blood was collected from the inferior vena cava before sacrifice [13]. In the study conducted by Kawasaki et al., the authors reported the same increase in blood sugar after anesthesia [63]. At week 18, insulin concentration remained similar among groups and within the same group over the study period. The consumption of a 40% fructose-enriched diet did not significantly affect insulin sensitivity, as evidenced by stable insulin concentrations [25,64]. Nonetheless, other studies have observed hyperinsulinemia in rats consuming a fructose-enriched diet (36% of TEI), indicating the presence of insulin resistance status [25,41]. Fructose contributes to an increase in insulin by interfering with the signaling of this hormone and by inhibiting the activity of the insulin receptors [25].

Elevated serum levels of ALT and AST are also markers of liver damage [25]. However, these values were within the normal range for rats between and within groups. Ferrere et al. found nonsignificant fluctuations when administering a diet enriched in fructose [64]. The ALT level is not strictly correlated with the severity of liver injury in NAFLD [64]. Genetic modifiers could also play a role in the resistance of these enzymes to disease progression in rats [25]. These results may suggest that a longer duration of the experimental study, a different rat strain, or a higher percentage of fructose may be necessary to significantly increase these markers in rats.

At week 18, the mean TG (mmol/L) was higher in the HFr group compared to the other groups, with a significant increase observed within the group (week 1–week 18). This finding is consistent with previous studies linking fructose consumption to dyslipidemia and fibrosis in rodents [13,15,37,41,44,48,65,66,67,68]. Fructose-induced hyperlipidemia can be attributed to the increase in de novo lipogenesis (DNL) and the expression of key lipogenic enzymes that stimulate the synthesis of nonesterified fatty acid (NEFA) in hepatocytes [41,44]. In the HFr-S5 and HFr-S10 groups, the increase in TG was not observed. Several studies have shown that spirulina, at different doses, improves hyperlipidemia [15,48,65,67]. In a recent study conducted by Hozayen et al., the administration of spirulina (50 mg/kg) significantly improved the lipid profile altered by a diet enriched in fructose (30%) [41]. Furthermore, El-Sheekh et al. found that the administration of different doses of spirulina (2.5%, 5%, or 10%) decreased hyperlipidemia in a dose-dependent manner [15]. Several explanations exist for the lipid-lowering role of spirulina. Spirulina contains C-phycocyanin, which binds to bile acids in the jejunum and inhibits jejunal absorption of cholesterol and ileal reabsorption of bile acids. Another suggestion is that the lipid-lowering effect of spirulina could be attributed to its richness in essential polyunsaturated fatty acids (omega-6 and omega-3) and niacin [19]. Moreover, spirulina may normalize TG levels by decreasing the production of very low-density lipoprotein (VLDL), enhancing its clearance in peripheral tissues, and stimulating the action of lipoprotein lipase [48,67].

Serum TNF-α and IL-6 levels were increased in patients with NAFLD and correlated with the histologic severity of liver damage [19,69,70]. In our study, the HFr group showed a stable level of TNF-α and IL-6 from week 1 to week 18. In contrast, TNF-α decreased in the HFr-S10 group over the same period. This aligns with a recent study that also observed similar results after administering spirulina to rats, which attributed these effects to spirulina’s antioxidant and anti-inflammatory properties [41]. It is mainly due to its richness in C-phycocyanin, β-carotene, vitamin E, phenolic compounds, and in ω-3 and ω-6 polyunsaturated fatty acids [19,41]. This alga also contains heptadecane, a volatile component that suppresses the expression of pro-inflammatory genes by reducing NF-κβ activity [16]. Thus, spirulina can decrease cell damage and play a role in the regeneration of damaged cells [17].

Adiponectin is an adipokine secreted exclusively by adipose tissue to stimulate insulin sensitivity, lipid oxidation, and anti-inflammatory effects [2,6]. The liver expression of the latter is reduced in animal models with NAFLD and indicates a failure in the oxidation of lipids, which contributes to their excessive accumulation in hepatocytes [36]. Similarly, serum adiponectin levels are reduced in patients with NAFLD [19,68,69]. In this study, the HFr group showed a decline in adiponectin levels (week 1–week 18). Fakhoury et al. and Hozayen et al. showed that rats consuming a fructose-enriched diet (30%) exhibited significantly low adiponectin levels [13,41]. However, the HFr-S5 and HFrS-10 groups did not exhibit a significant difference in mean adiponectin levels between the two time periods, suggesting that spirulina may mitigate the adverse effects of fructose on adiponectin levels. In a recent study, rats provided with spirulina exhibited identical outcomes [41]. It could be explained by the antioxidant and anti-inflammatory properties previously mentioned [16,19,41]. It was also speculated that the suppression of TNF-α release following the administration of spirulina could be a direct result of increased serum adiponectin levels [41].

Serum urea, a kidney parameter, decreased in the HFr-S10 group at week 1 and week 18 (Table 5). Similar results were shown in other studies [53,54]. Spirulina reduces kidney damage by improving indicators of renal function [53,54]. It also enhances antioxidant enzymes and inhibits lipid peroxidation, which is responsible for initiating and developing nephrotoxicity [53,54].

According to the phylae composition, and for the same period, the HFr group exhibited a significant increase in the *Firmicutes*/*Bacteroidetes* ratio compared to the HFr-S5 and HFr-S10 groups. This ratio has been correlated with an increase in obesity, type 2 diabetes, metabolic syndrome, and NAFLD [71,72]. The development of these chronic diseases could be result of bacterial translocation and secretion of endotoxins such as *Staphylococcus and Enterococcus* related to a fructose-enriched diet [70,72]. Likewise, at week 18, the abundance of the *Fibrobacteria* phylae, known for maintaining the balance of IM, increased significantly in the HFr-S10 group, correlating with spirulina’s antioxidant and anti-inflammatory properties [25], as well as its richness in C-phycocyanin, vitamin E, phenolic compounds, and ω-3 and ω-6 polyunsaturated fatty acids capable of reducing the fructose inflammatory effect [24,25].

At the family level, and for the same period, the decline in *Prevotellaceae* in both HFr-S5 and HFr-S10 groups was less compared to the HFr group, with the HFr-S10 group demonstrating a similar abundance to the CT group. This family is known for its production of SCFA, such as propionate and acetate as well as thiamine and folate [73]. This result was consistent with the results obtained by Chandrarathna et al. [73]. The spirulina with its anti-inflammatory effects has been shown to neutralize the effect of fructose by increasing the number of *Prevotellaceae* and subsequently decreasing the inflammation induced by the fructose-enriched diet [73]. Similarly, and for the same period, the *Lactobacillaceae* family exhibited a notable decrease in the HFr group, which was significantly different from the CT group. A study by Kulshreshtha et al. showed that *Lactobacillaceae* are lactic acid bacteria that can inhibit pathogens, improve intestinal barrier function, modulate immune responses, and subsequently alter the natural history of NAFLD [74]. The diets enriched with spirulina (*Arthrospira platencis*) at different percentages could have alleviated HFr-induced oxidative damage and intestinal tissue inflammation and consequently maintained *Lactobacillaceae* levels in the gut [75]. Furthermore, it seems that spirulina (*Arthrospira platencis*) has a stimulatory effect on the growth and survival of lactic acid bacteria such as *Lactobacillus thermophilus*, *Lactobacillus acidophilus*, and *Lactobacillus bulgaricus* [76]. These bacteria are largely used as probiotics and as a starter for yogurt production [76]. The phenolic compounds present in spirulina exert antimicrobial activities and improve the growth of probiotics [76].

This study showed that the abundance of the *Ruminococcaceae* family increased significantly at week 18 in the HFr group, compared to the HFr-S10 group. Studies reported that some species of the genus *Ruminococcus* (*R. torques*, *R. gnavus*) are proinflammatory and capable of producing ethanol, two potential pathogenic mechanisms in the progression of NAFLD [73,74]. The abundance of *Ruminococcus gravus* and *R. torques* may contribute to the pathogenesis of IBD by providing a substrate to sustain non-mucolytic mucosa–associated bacteria [77].

The diversity of the composition of IM was assessed at two levels as follows: alpha and beta diversity. At week 1, there was no variation in alpha diversity between the groups (*p* > 0.05), because the four groups of rats had the same richness and diversity. This could be explained by the fact that the rats came from the same breeding and were almost the same age. However, a significant difference in alpha diversity was seen within each group at the two time periods (*p* < 0.05). The alpha diversity was lower in all groups at week 18 compared to week 1, indicating less diversity and less bacterial richness. This can be due to the excessive weight gain in all rats in the different groups and the advancement in age [78]. At week 18, a significant disparity in alpha diversity between the groups was observed. The HFr group has less diversity and richness than the other groups, and more dysbiosis caused by this type of diet. Thus, spirulina has a favorable influence on the composition of the IM, facilitating the restoration of the bacterial diversity. Results are relevant because the HFr-S group had even better diversity than the CT group. This result was confirmed by the beta diversity (Unifrac model), which demonstrated a similarity between the HFr-S5 and HFr-S10 groups, because the microbiota profiles cluster together and form a distinct cluster separate from the CT and the HFr groups. The two groups enriched in spirulina had the same richness and the same bacterial diversity at week 18.

This study has several limitations such as measurement biases as well as in biological and microbiological analyses. Pancreatic parameters were not considered in this research owing to the small amount of blood collected at weeks 1 and 18. Moreover, the biochemical analysis of the different parameters was not reported at different time points due to related complications, such as glycemic changes, respiratory distress, or mortality risks. Furthermore, the pathway mechanism of the effects of spirulina on organ lesions induced by a high-fructose diet should be further investigated at the molecular and cellular levels.

## 5. Conclusions

We conclude that administering 5% to 10% of spirulina to a fructose-enriched diet may maintain serum triglycerides and adiponectin levels. A dose of 10% of spirulina to this diet may decrease liver fat weight, serum TNF-α, and urea levels. Furthermore, adding 10% spirulina to this fructose-enriched diet rebalanced IM by reincreasing the *Prevotellaceae* and *Lactobacillaceae* family and decreasing the ratio of *Firmicutes:Bacteroidetes*. The spirulina was also able to rebalance the harmful effect of fructose on IM by maintaining its richness and the bacterial diversity of the latter. Nevertheless, spirulina had slight effects on liver necroinflammation, portal, and perisinusoidal fibrosis as well as on renal and pancreatic inflammation and fibrosis. Further experimental studies are needed to ascertain the effect of spirulina on the liver, kidneys, pancreas, and IM in the long term, especially since few studies have been based on histopathology results. The time and the dose of the administered spirulina should be further studied. These findings may open the door for the development of new strategies for targeted intervention to prevent or treat hepatic, pancreatic, and renal diseases associated with NAFLD as well as maintain the diversity and composition of the IM.

## Figures and Tables

**Table 1:** Ingredient composition of the four diets fed to the rats (week 2–week 18).

Ingredients	CT Diet 17% Fat, 20% Proteins, 62% Carbohydrates		HFr Diet 20% Fat, 20% Proteins, 60% Carbohydrates (40% Fructose)		HFr-S5 Diet 20% Fat, 20% Proteins, 60% Carbohydrates (40% Fructose), Spirulina 5% of the Total Weight		HFr-S10 Diet 20% Fat, 20% Proteins, 60% Carbohydrates (40% Fructose), Spirulina 10% of the Total Weight	
	(g)	(kJ) ^(3)^	(g)	(kJ)	(g)	(kJ)	(g)	(kJ)
Casein	200	2995.7	200	2995.7	200	2995.7	200	2995.7
DL-methionine	2	33.5	2	33.5	2	33.5	2	33.5
Corn starch	530	7986.9	92.5	1393.9	92.5	1393.9	92.5	1393.9
Sucrose	100	1590.7	119.8	1905.6	119.8	1905.6	119.8	1905.6
Fructose	0	0	400	6362.7	400	6362.7	400	6362.7
Cellulose, BW ^(1)^	50	0	50	0	50	0	50	0
Mineral mix ^(2)^	35	129	35	129	35	129	35	129
Soybean oil	70	2636	70	2636	70	2636	70	2636
Butter	0	0	19	636.3	19	636.3	19	636.3
Vitamin mixture ^(2)^	10	162	10	162	10	162	10	162
Choline Bitartrate	2	0	2	0	2	0	2	0
Total	999	15,534	1000	16,255	1000	16,255	1000	16,255
Spirulina	0		0		50		100	

^(1)^ Alpha-cellulose grade BW-200. Maple Biotech Pvt. Ltd., Pune, Maharashta, India. ^(2)^ Composition of the vitamin mixture (AIN-93-VX; Clea, Tokyo, Japan) and mineral mixture (AIN-93G-MX; Clea, Tokyo, Japan) [18]. ^(3)^ Energy values (kJ) of the corresponding items (g) (energy values were calculated according to the typical caloric density values for commonly used dietary ingredients for laboratory animals; Dyets, Inc., Bethlehem, PA, USA). CT, control diet; HFr, high-fructose diet; HFr-S5, high-fructose diet and spirulina (5%); HFr-S10, high-fructose diet and spirulina (10%).

**Table 2:** Body weights, food energy intakes, food consumed, and organ weights (liver and kidney).

	CT Group	HFr Group	HFr-S5 Group	HFr-S10 Group
Rats body weight (g)				
Week 1	209.5 ± 0.03 ^NS^	216.4 ± 0.09	235.1 ± 0.04	219.2 ± 0.06
Week 18	405.3 ± 0.03 ^§^	414.1 ± 0.09 ^§^	425.7 ± 0.04 ^§^	408.9 ± 0.03 ^§^
Energy intake (kJ/week)				
Week 2	1980.1 ± 0.05 ^NS^	1884.9 ± 0.07	2025.8 ± 0.07	1884.1 ± 0.09
Week 18	1951.2 ± 0.05 ^NS^	2078.2 ± 0.05 ^§^	1816.3 ± 0.06	1778.6 ± 0.05
Approximate amount consumed (g/week)				
Week 2	126.7 ± 0.05 ^NS^	115.2 ± 0.07	123.8 ± 0.07	115.2 ± 0.09
Week 18	124.8 ± 0.05	127.0 ± 0.05 ^§^	111.0 ± 0.06	108.7 ± 0.05
Weight gain (g)				
Week 18–Week 1	195.3 ± 26.6 ^NS^	196.6 ± 37.6	191.45 ± 37.11	188.79 ± 26.19
Liver weight (g)				
Week 18	9.5 ± 0.03 ^b^	10.8 ± 0.04 ^a^	10.0 ± 0.05	9.7 ± 0.03
Liver (g)/100 g of body weight				
Week 18	2.3 ± 0.1 ^NS^	2.6 ± 0.2	2.1 ± 0.7	2.1 ± 0.7
Kidney weight (g)				
Week 18	1.5 ± 0.05 ^NS^	1.6 ± 0.08	1.5 ± 0.06	1.5 ± 0.02
Epididymal fat weight (g)				
Week 18	7.1 ± 0.14 ^NS^	7.2 ± 0.21	6.8 ± 0.20	5.8 ± 0.10
Pancreas weight (g)				
Week 18	0.9 ± 0.30 ^NS^	0.8 ± 0.30	0.7 ± 0.10	0.6 ± 0.10
Liver lipid weight (mg)				
Week 18	41.44 ± 0.14	50.11 ± 0.19 ^c^	32.42 ± 0.21	28.47 ± 0.13 ^b^
Renal lipid weight (mg)				
Week 18	69.44 ± 0.20 ^NS^	57.0 ± 0.32	71.8 ± 0.33	45.2 ± 0.31

Data are means ± SD, n = 10 rats/group. Geometric means (Log10 of continuous variables) were used in the case of nonnormality of the distribution. CT, control diet; HFr, high-fructose diet; HFr-S5, high-fructose diet and spirulina (5%); HFr-S10, high-fructose diet and spirulina (10%). “^a,b,c^” refer to differences between groups (*p* < 0.05). ^a^ refers to significant differences with group CT. ^b^ refers to significant differences with group HFR. ^c^ refers to significant differences with group HFR-S10. ^§^ corresponds to a significant variation within the same group between time 1 and time 2 (*p* < 0.05). ^NS^, not significant between groups. One-way ANOVA and paired *t*-tests between groups and within groups over week 1 and week 18 for normally distributed data were also performed followed by the Bonferroni multiple comparisons test.

**Table 3:** Pathology features of the rat livers in the four groups at week 18.

	CT Group	HFr Group	HFr-S5 Group	HFr-S10 Group
Steatosis (%) ^(1)^	8.75 ± 0.10 ^NS^	10.67 ± 0.24	10.51 ± 0.16	1.33 ± 0.13
Microvesicular ^(2)^				
None	4	3	2	6
+	5	3	7	4
++	1	4	1	0
Macrovacuolar ^(2)^				
None	4	4	4	7
+	6	4	5	3
++	0	2	1	0
Necroinflammation ^(3)^				
0	5	2	3	5
1	5	8	6	4
2	0	0	1	1
Portal fibrosis ^(3)^				
0	10	9	8	9
1	0	1	2	1
2	0	0	0	0
Perisinusoidal fibrosis ^(3)^				
0	9	9	9	8
1	1	1	1	2
2	0	0	0	0

^(1)^ Results for steatosis are means ± SD. ^NS^ not significant between groups. CT, control diet; HFr, high-fructose diet; HFr-S5, high-fructose diet and spirulina 5%; HFr-S10, high-fructose diet and spirulina 10%. Geometric means (Log 10 of continuous variables) were used in the case of nonnormality of the distribution. One-way ANOVA between groups was performed for normally distributed data, followed by the Bonferroni multiple-comparisons test. ^(2)^ Results for macrovacuolar and microvesicular steatosis are given as the number of rats presenting mild+ (<33% of hepatocytes) or moderate++ (33–66% of hepatocytes). ^(3)^ Necroinflammation and fibrosis are given as the number of rats presenting a score of 0 (absent), 1 (mild), or 2 (moderate), respectively.

**Table 4:** Pathology features of the kidneys and pancreas in the four groups of rats at week 18.

	CT Group	HFr Group	HFr-S5 Group	HFr-S10 Group
Renal inflammation ^(1)^				
0	6	4	9	3
1	4	6	1	6
2	0	0	0	1
Glomerulosclerosis ^(1)^				
0	10	10	10	10
1	0	0	0	0
2	0	0	0	0
Interstitial renal fibrosis ^(1)^				
0	7	7	7	5
1	3	3	3	5
2	0	0	0	0
Pancreatic inflammation ^(1)^				
0	9	6	9	8
1	1	4	1	2
2	0	0	0	0
Pancreatic tissue fibrosis ^(1)^				
0	7	2	8	5
1	3	8	2	5
2	0	0	0	0
Anomaly of the islets of Langerhans ^(2)^				
0	10	10	10	10
1	0	0	0	0

CT, control diet; HFr, high-fructose diet; HFr-S5, high-fructose diet, and spirulina 5%; HFr-S10, high-fructose diet and spirulina 10%. n = 10 rats/group. ^(1)^ Results for renal and pancreatic inflammation are given as the number of rats presenting a score of 0 (absence), 1 (mild), or 2 (moderate). Results for interstitial renal fibrosis, glomerulosclerosis, and pancreatic tissue fibrosis are given as the number of rats presenting a score of 0 (absence) or 1 (presence). ^(2)^ Results for the pancreatic islets of Langerhans (hyperplasia or hypertrophy) are given as the number of rats presenting a score of 0 (absence) or 1 (presence).

**Table 5:** Comparison of the rat serum chemistries among and within the four groups (week 1 and week 18).

	CT Group	HFr Group	HFr-S5 Group	HFr-S10 Group
Glucose (mmol/L)				
Week 1	7.77 ± 0.14 ^NS^	6.83 ± 0.11	6.11 ± 0.20	6.09 ± 0.18
Week 18	5.19 ± 0.05 ^§bcd^	8.59 ± 0.07 ^a^	7.23 ± 0.11 ^a^	7.36 ± 0.12 ^a^
Triglycerides (mmol/L)				
Week 1	0.46 ± 0.11 ^NS^	0.46 ± 0.10	0.59 ± 0.13	0.62 ± 0.16
Week 18	0.62 ± 0.10 ^§b^	0.94 ± 0.13 ^§acd^	0.62 ± 0.08 ^b^	0.62 ± 0.08 ^b^
Insulin (pmol/L)				
Week 1	28.46 ± 0.28 ^NS^	46.74 ± 0.35	31.88 ± 0.30	39.91 ± 0.41
Week 18	31.10 ± 0.18 ^NS^	39.19 ± 0.38	25.6 ± 0.19	33.26 ± 0.11
TNF-α (pg/mL)				
Week 1	28.24 ± 0.15 ^d^	26.74 ± 0.05 ^d^	29.65 ± 0.10	39.79 ± 0.12 ^ab^
Week 18	27.70 ± 0.12 ^NS^	30.30 ± 0.13	28.40 ± 0.15	27.08 ± 0.14 ^§^
IL-6 (pg/mL)				
Week 1	30.61 ± 0.14 ^c^	31.17 ± 0.09	43.16 ± 0.09 ^a^	41.10 ± 0.11
Week 18	33.09 ± 0.17 ^NS^	33.54 ± 0.15	38.99 ± 0.09	40.84 ± 0.16
Adiponectin (ng/mL)				
Week 1	11.59 ± 0.05 ^NS^	11.81 ± 0.06	11.52 ± 0.06	11.37 ± 0.06
Week 18	10.88 ± 0.06 ^§NS^	10.61 ± 0.09 ^§^	11.21 ± 0.08	11.10 ± 0.08
ALT (UI/L)				
Week 1	26.53 ± 0.08 ^NS^	24.09 ± 0.15	21.45 ± 0.07	18.44 ± 0.18
Week 18	21.25 ± 0.14 ^NS^	19.50 ± 0.08	19.18 ± 0.14	24.57 ± 0.11
AST (UI/L)				
Week 1	28.18 ± 0.05 ^bd^	22.26 ± 0.08 ^a^	23.39 ± 0.11	21.21 ± 0.07 ^a^
Week 18	26.99 ± 0.11 ^NS^	25.21 ± 0.17	26.44 ± 0.14	21.81 ± 0.24
Creatinine (μmol/L)				
Week 18	81.94 ± 0.08 ^b^	52.29 ± 0.11 ^a^	62.65 ± 0.13	62.63 ± 0.07
Urea (mmol/L)				
Week 1	13.66 ± 0.09 ^NS^	15.08 ± 0.08	13.55 ± 0.06	17.4 ± 0.12
Week 18	12.64 ± 0.12 ^NS^	12.32 ± 0.12	12.07 ± 0.11	12.49 ± 0.09 ^§^

Data are means ± SD, n = 10 rats/group. ^NS^, not significant between groups. CT, control diet; HFr, high-fructose diet; HFr-S5, high-fructose diet and spirulina (5%); HFr-S10, high-fructose diet and spirulina (10%). “^a,b,c,d^” refer to differences between groups (*p* < 0.05). ^a^ refers to significant differences with group CT. ^b^ refers to significant differences with group HFR. ^c^ refers to significant differences with group HFR-S5. ^d^ refers to significant differences with group HFR-S10. ^§^ corresponds to a significant variation within the same group between week 1 and week 18 (*p* < 0.05).

## Data Availability

Data are contained within the article and can be provided upon request.

## Abbreviations

ALT	alanine aminotransferase
ARRP	Animal Research Review Panel
AST	aspartate aminotransferase
CT	control
DNL	de novo lipogenesis
GI	glycemic index
GNC	general nutrition care
HFr	high-fructose diet
HCC	hepatocellular carcinoma
H&E	hematoxylin and eosin
HFr-S5	high-fructose 5% spirulina
HFr-S10	high-fructose 10% spirulina
IL-6	interleukine-6
IM	intestinal microbiota
IR	insulin resistance
MASH	metabolic dysfunction–associated steatohepatitis
MASLD	metabolic dysfunction–associated steatotic liver disease
NAFLD	nonalcoholic fatty liver disease
NASH	nonalcoholic steatohepatitis
NEFA	nonesterified fatty acid
OTU	operational taxonomic unit
ROS	reactive oxygen species
SCFA	short-chain fatty acids
SD	standard deviation
T2DM	type 2 diabetes mellitus
TEI	total energy intake
TG	triglycerides
TNF-α	tumor necrosis factor
USA	United States of America
VLDL	very low-density lipoprotein
